# Supplementary material for: Are carbon emissions trading and green financial instruments synergistic? -Comprehensive quantitative research based on content analysis
Source: PLoS One. 2024 Mar 7;19(3):e0298601. doi: 10.1371/journal.pone.0298601 (PMC10919601; doi:10.1371/journal.pone.0298601)
Supplement: S1 Data — (ZIP) [file pone.0298601.s001.zip › Data/地级市Z_F工作报告.zip!/║■─╧/╥μ╤⌠╩╨/└┤╫╘╣1⁄2╓┌║┼í╛┬φ┐╦╩2╛▌═°í┐_2009.docx]

各位代表：

现在，我代表市人民政府，向大会作政府工作报告，请予审议，并请各位政协委员和其他列席人员提出意见。

一、关于过去五年的工作

过去五年，在中共益阳市委的正确领导下，全市人民坚持以邓小平理论和“三个代表”重要思想为指导，深入贯彻落实科学发展观，扎实工作，开拓进取，经济社会发展取得了显著成就。

过去五年，经济发展提速增效。发展速度明显加快。2008年完成生产总值511.28亿元，2003-2008年年均增长12.4%；完成财政总收入30.08亿元，年均增长21.5%；完成全社会固定资产投资258.22亿元，年均增长32.8%；实现社会消费品零售总额188亿元，年均增长15.7%。经济结构不断优化。三次产业比由26.5︰28.0︰45.5调整为28.4︰33.4︰38.2。2008年工业增加值占生产总值的比重达到29.3%，比2003年提高6个百分点，高新技术产业增加值占规模工业增加值的比重达到24%，提高18.7个百分点；规模工业企业达到629家，比2003年增加225家，产值过亿元的企业由2003年的12家增加到82家，其中过10亿元的4家。装备制造、食品、电力、化工、有色金属冶炼、纺织等主导产业初具规模。资水梯级开发取得重大进展。旅游等现代服务业加快成长。发展质量全面提升。2008年财政总收入占生产总值的比重比2003年提高1.09个百分点。规模工业经济效益综合指数达到198.98%，比2003年提高108.38个百分点。金融机构年末各项存款余额425.02亿元，各项贷款余额207.52亿元，分别是2003年末的2.2倍、1.4倍。

过去五年，城乡建设步伐加快。累计完成全社会固定资产投资751亿元。基础设施建设成效显著。益沅一级公路、茅草街大桥等项目建成通车，完成县到乡镇公路建设686公里，完成农村公路通畅通达工程6118公里。实施各类水利工程18.5万个。新建和改造35千伏以上变电站24座、10千伏以上线路1470公里，农村电网改造取得成效，赫山区新农村电气化示范县建设全面完成。新农村建设扎实推进，5个示范片、102个示范村建设取得明显成效。投入资金4.3亿元，实施土地整理28.5万亩。城镇化水平不断提高。完成了《益阳市2004－2020年城市总体规划》的调整工作。建成了团洲污水处理厂，实施了益阳大道、秀峰公园的提质改造。中心城区建成区面积由41平方公里扩大到50.5平方公里，人口由41.2万人增加到51万人。全市城镇化率由29.49%提高到39.75%。

过去五年，改革开放深入推进。国企改革不断深入，全市已有425家国有企业完成改革，占国有企业总数的81%。国有资产监管工作得到加强。农村综合改革取得重大成效。财税、教育、文化、卫生体制改革稳步推进，人事、行政审批、收入分配、集体林权等制度改革进展顺利。对外开放不断扩大。累计利用市域外资金706.7亿元，年均增长53.25%；其中直接利用外资2.46亿美元，年均增长27.67%。引进世界500强、国内500强、中央企业和知名上市公司等38家。成功承办了第三届湘台经贸论坛。累计完成进出口总额8.84亿美元，年均增长19%；其中出口5.88亿美元，年均增长42.4%。益阳被确定为国家加工贸易梯度转移重点承接地。

过去五年，社会事业全面进步。实施国家和省级重点科技项目185个，取得各类科技成果158项，科技进步对经济增长的贡献率达到46%。成功举办了益阳市首届产学研对接和科技成果交易会。各级各类教育持续发展，财政性教育投入占生产总值的比例由2.78%提高到3.45%。教育资源整合取得明显成效。教育教学质量居全省前列。文化事业日益繁荣。安化千两茶工艺和益阳茯砖茶手筑工艺被确定为国家非物质文化遗产代表作。广播电视事业实现跨越式发展，综合覆盖率达到98%。疾病预防控制体系和突发公共卫生事件应急机制初步建立，农村和社区卫生服务体系逐步完善。农村药品“两网”建设稳步推进。益阳被确定为国家羽毛球队训练基地，连续承办了第十九届、第二十届世界杯羽毛球赛。加强人口计生工作目标管理，连续五年人口出生率控制在12‰以内，人口自然增长率稳定在5‰以下。国土和矿产资源管理加强，土地市场进一步规范，重大项目用地需求基本得到保障。坚持污染防治与生态保护并重，环境质量得到改善。人民防空工作取得新成效。价格调控监管工作得到加强。国税、地税、审计、工商、质监、保险、档案、统计、民族、宗教、外事、侨务、对台、科协、老龄、残疾人、地方志、地震、气象、水文、无线电管理等工作都取得新的成效。

过去五年，人民生活持续改善。2008年城镇居民人均可支配收入12448元，农民人均纯收入4526元，年均分别增长12.6%、13.6%。全面取消了农业税和农业特产税，累计发放各种惠农补贴资金11.58亿元。农村义务教育“两免一补”政策得到落实。坚持为民办实事，解决了一批事关群众切身利益的实际问题。新增城镇就业20.8万人，国企下岗职工11万多人实现再就业，在册零就业家庭全部得到就业援助，城镇登记失业率控制在4.5%以内。全市转移劳动力111万多人，比2003年增加46万多人，劳务收入达到100亿元。企（事）业养老、医疗、失业、工伤、生育保险覆盖面不断扩大，保障水平明显提高。城乡低保、医疗救助、救灾救济、慈善救助等各项工作稳步推进，廉租房、农村安居工程、城镇经济适用房、敬老院建设和住房公积金工作等成效显著。扶贫开发整村推进顺利实施，全市贫困人口由2003年的34.3万人下降到20.2万人。

过去五年，社会保持和谐稳定。精神文明和民主法制建设不断加强。深入开展了保持共产党员先进性教育和解放思想大讨论活动。广泛开展文明创建活动，社会文明程度明显提高。认真执行人大及其常委会的决议、决定，自觉接受人大的法律监督、工作监督和政协的民主监督。共办理省市人大代表建议708件、省市政协委员提案941件。认真贯彻实施行政许可法，依法行政全面推进。反腐倡廉、纠风治乱工作取得阶段性成果。安全生产形势持续好转。强化维稳工作责任，加强社会治安综合治理，切实做好信访工作，社会政治大局保持稳定。

国防动员和后备力量建设得到加强。人民解放军、武警部队、预备役部队和民兵，在支持地方经济建设、抗灾抢险等方面作出了重要贡献。积极推进拥军优属、拥政爱民，军政军民关系更加密切。

2008年，我们战胜历史罕见的低温雨雪冰冻灾害，沉着应对国际金融危机冲击，积极支援地震灾区恢复重建，大力支持北京成功举办奥运会和残奥会，经济社会持续较快发展。突出抓了五项工作。一是大力推进新型工业化。全年完成工业增加值149.78亿元，比上年增长20.6%，其中规模工业增加值127.92亿元，增长24.9%。工业增加值在全省的比重达到3.5%，比上年提高0.6个百分点。万元生产总值能耗和万元规模工业增加值能耗分别下降4.2%和10.8%。化学需氧量和二氧化硫排放量分别下降4.2%和4.4%。二是大力发展现代农业。优质稻米、林纸板材、棉麻纺织、水产畜禽、油蔬茶果五大农业产业链基地面积稳定在1000万亩以上，无公害、绿色、有机农产品产地认定面积达到385万亩。粮食总产量、生猪出栏数、黑茶产值分别增长8.5%、23.7%和37%。现代林业示范市建设成效明显。实现农产品加工增加值45亿元，比上年增长27.5%；市级以上农业产业化龙头企业达到174家。三是大力推进新型城镇化。加快了高新区东部新区规划编制工作。完成了龙洲路、金山南路等道路改造工程。加强城市管理，市容市貌得到改观。加大城市资源优化配置和经营开发力度，完成城建融投资10.1亿元。加快小城镇发展，完成小城镇基础设施建设投资3.97亿元。四是大力加强招商引资和项目建设。全年利用市域外资金246.6亿元，比上年增长26.46%，其中直接利用外资8000万美元，增长33.4%。投资600亿元的桃花江核电项目落户益阳。重点项目建设进展顺利，益阳电厂二期、柘溪电站扩容、益阳东220千伏变电站、中联重科搅拌机沅江基地、晶鑫新能源一期、龙建达二期、益华水产加工城等项目竣工投产，益宁城际干道、凯迪生物质能热电等项目正在加快建设。启动了益阳绕城高速和二广高速桃马联络线的建设。五是大力实施“三大工程”。“民生工程”考核项目和省为民办实事项目全面完成，“城乡清洁工程”全面实施，“平安工程”建设扎实推进。

各位代表！过去五年取得的成绩，是在中共益阳市委的坚强领导下，全市人民团结奋斗的结果。在此，我代表市人民政府，向全市广大工人、农民、干部和知识分子，向各民主党派、工商联、无党派人士、各人民团体和社会各界人士，向驻益人民解放军和武警部队官兵、政法干警，向中央、省属驻益单位，向热心支持益阳改革发展的各级领导和各界朋友，表示衷心的感谢！

在肯定成绩的同时，应该清醒地看到，我们的工作与人民的期待还有差距。一是经济整体实力不强，工业化水平不高，结构性矛盾仍然比较突出，节能减排任务艰巨；二是农业基础设施薄弱，农民持续增收难度加大；三是部分群众生活比较困难，就业再就业压力大，影响社会和谐稳定的因素仍然较多；四是政府自身建设需要进一步加强，经济发展环境有待进一步优化；等等。这些问题，我们将采取切实有效的措施认真加以解决。

二、关于今后五年的工作

做好未来五年的工作，我们必须认清形势，正视挑战，把握机遇，坚定信心，奋发有为，努力开创各项工作新局面。

当前，世界经济金融形势复杂多变，不稳定不确定因素明显增多。次贷危机引发的金融危机愈演愈烈，迅速从局部发展到全球，从发达国家传导到新兴市场国家和发展中国家，从金融领域扩散到实体经济领域，酿成了一场历史罕见、冲击力极强、波及范围很广的国际金融危机。在国际金融危机冲击下，实体经济增速大幅下滑。目前，这场金融危机不仅本身尚未见底，而且对实体经济的影响正进一步加深，其严重后果还会进一步显现。目前我市经济运行中的困难增加，经济下行压力加大，企业经营困难增多，保持农业稳定发展、农民持续增收难度加大。去年下半年以来，我市生产总值、财政收入、全社会固定资产投资等主要经济指标增幅明显回落。近年来，我市经济发展速度加快，效益提高，但无论是经济总量还是人均指标，与全省平均水平比，都有较大差距。要实现市第四次党代会提出的综合实力进入全省第二方阵的目标，任务十分艰巨。

在清醒认识面临的严峻形势的同时，我们更要看到加快发展的有利条件。我市现阶段的发展主要依靠投资拉动，国家扩内需、保增长的宏观政策对我们整体有利，只要我们审时度势，用好、用活、用足国家政策，充分发挥自身优势，完全可以化挑战为机遇，争取更多的项目和资金支持。我们多年扎实打基础的工作开始显现成效，蓄积的发展能量开始释放；国际国内产业转移步伐加快，有利于我们进一步扩大招商引资；一批重大基础设施开工建设，一批战略投资者相继进驻，一批重大产业项目落户，为加快发展提供了有力支撑。全市上下加快发展的认识更加统一，思路更加清晰，措施更加有力，只要我们团结带领全市人民共同奋斗，肩负起加快发展的神圣使命，完全可以实现科学发展、后发赶超。

今后五年经济社会发展的总体要求是：全面贯彻党的十七大和十七届三中全会精神，坚持以邓小平理论和“三个代表”重要思想为指导，深入贯彻落实科学发展观，紧紧围绕市第四次党代会提出的目标，继续解放思想，不断改革创新，坚持以新型工业化为主导，切实加强基础设施、基础产业和基础工作，大力推进新型城镇化和以现代农业为核心的社会主义新农村建设，突出抓好招商引资和项目建设，突出加强城乡统筹，突出改善民生，努力建设富强益阳、绿色益阳、开放益阳、和谐益阳。

——加快富民强市步伐，生产总值年均增长13%以上，财政总收入年均增长20%以上，全社会固定资产投资年均增长30%以上，社会消费品零售总额年均增长15%以上，城镇居民人均可支配收入年均实际增长10%以上，农民人均纯收入年均实际增长10%以上。到2013年，力争全市生产总值突破1000亿元，财政总收入达到80亿元，综合实力进入全省第二方阵。经济结构不断优化，工业经济比重提高到38%以上。城乡区域协调发展，城镇化率达到46%以上，社会主义新农村建设取得新的进展。科技进步对经济增长的贡献率达到55%。不断增强我市的经济实力，不断提高人民群众的富裕程度和幸福指数，努力建设富强益阳。

——按照两型社会的要求，努力建设新型工业城市、宜居山水生态城市，积极创建国家森林城市、中国优秀旅游目的地城市、国家卫生城市、国家环境保护模范城市，万元生产总值能耗和万元规模工业增加值能耗低于全省平均水平，主要污染物排放达到国家规定标准，森林覆盖率达到52.5%以上，大型水体水质达到功能区要求，努力建设绿色益阳。

——优化发展环境，放活投资主体，放开投资领域，放宽市场准入，激活各类生产要素，促进多种所有制经济共同发展。力争引进市域外资金年均增长20%以上，进出口总额年均增长20%以上，其中出口年均增长30%以上。树立世界眼光和战略思维，加快融入长株潭，有效对接长三角、珠三角，加强对外交流合作，构建全方位、多层次、宽领域的大开放格局，努力建设开放益阳。

——不断完善利益调节机制和社会保障体系，着力解决教育、就业、医疗卫生等关系人民群众切身利益的问题。继续稳定低生育水平，人口出生率控制在12.5‰以内，人口自然增长率控制在6‰以内。力争创建省级、国家级“双拥模范城”。不断提高城乡文明程度，加强民主法制建设，社会公平正义得到充分体现，社会治安综合治理进一步加强，社会大局保持稳定，努力建设和谐益阳。

实现上述目标，必须牢牢把握以下原则：

一是必须继续解放思想。坚持与时俱进，突破各种思想障碍和条条框框，大力推进观念创新、制度创新、管理创新、方法创新，大胆探索促进科学发展的新思路、新举措，力求在操作上突破，在细节上突破，在落实上突破，着力构建有利于科学发展、充满生机活力的体制机制，使我们的工作体现时代性、把握规律性、富于创造性。

二是必须着力夯实基础。坚持把加强“三个基础”作为提升发展水平、支撑发展全局的重大战略措施，充分发挥基础设施的支撑作用、基础产业的带动作用、基础工作的保障作用，不断增强发展后劲。

三是必须奋力后发赶超。紧紧围绕科学发展、后发赶超目标，对照先进，瞄准一流，以只争朝夕的精神，以积极有为的姿态，以敢为人先的气魄，打破常规开展工作，不动摇，不懈怠，不折腾，抢抓机遇，加快发展，力争早日跻身全省第二方阵。

四是必须坚持以人为本。坚持一切工作以人民利益为根本价值取向，以民生改善为最终检验标准，更加注重富民惠民，千方百计改善民生，让人民群众共享改革发展成果，使全市人民学有所教、劳有所得、病有所医、老有所养、住有所居。

三、关于2009年的工作

2009年是新一届政府的开局之年，我们一定要团结一心，知难而进，顽强拼搏，突出保增长、扩内需、调结构、促就业、强基础。经济社会发展的主要预期目标是：生产总值增长11%以上，财政总收入增长12%以上，全社会固定资产投资增长35%以上，社会消费品零售总额增长15%，城镇居民人均可支配收入实际增长9%，农民人均纯收入实际增长9%，城镇登记失业率控制在4.8%以内，居民消费价格涨幅控制在4%以内，万元生产总值能耗下降4%，万元规模工业增加值能耗下降5.2%，化学需氧量和二氧化硫排放量分别控制在8.8万吨和7.2万吨以下，人口自然增长率控制在6‰以内。重点抓好九项工作。

（一）千方百计扩大投资，增强经济发展后劲

大力抓好争资立项工作。把争资立项作为落实宏观调控政策、促进加快发展的重要举措。认真研究国家扩大投资的方向和重点，超前谋划、积极对接，围绕基础设施、民生工程、生态环境建设、产业结构调整等重点领域，精心策划包装一批大项目、好项目。积极向上汇报，力争有更多项目进入国家和省里计划笼子。尽快启动一批既能拉动当前经济增长、又能增强经济发展后劲的重大基础设施和产业发展项目。

进一步加大招商引资力度。始终坚持把招商引资作为加快发展的战略选择和根本举措。认真研究招商引资面临的新形势，不断创新招商引资方法，大力推进以商招商、专业招商、小分队招商和委托招商。实行战略资源、优惠政策、政治荣誉向战略投资者倾斜，大力引进战略投资者。以加快承接产业转移为突破口，大力推进工业招商，力争工业项目到位资金增长30%以上。充分发挥加工贸易梯度转移重点承接地的政策优势，突出抓好加工贸易产业招商，立足现有资源和基础，以益阳高新区和各区县（市）工业园区为重点，打造优势承接平台，力争在集群式承接上取得突破。大力优化投资环境，严格执行“十不准”，坚决执行投资项目全程代理服务制度，开辟重大投资项目绿色通道。抓好签约项目的跟踪调度，提高项目履约率、资金到位率和开工投产率。进一步完善招商引资目标管理责任制，对市直各部门和各区县（市）招商引资工作实行严格考核。

鼓励引导金融机构支持地方经济发展。抓好社会信用体系建设，改善金融生态环境，促进政府与银行、企业与银行沟通合作，努力缓解企业融资难问题。鼓励引导金融机构加大对工业、农业、基础设施和服务业等重点领域的信贷资金投放。支持金融创新。支持桃江建信村镇银行发展。全力支持有条件的企业尽快上市，努力培育后备上市资源。积极探索发行企业债券、股权融资等直接融资方式。加强信用担保体系建设。健全保险市场体系和服务体系，发挥保险的社会保障功能。

努力扩大民间投资。放宽民间资本市场准入，挖掘民间资本投资潜力，鼓励民间资本投向基础设施、公用事业和其他行业领域。千方百计激发民间投资热情，鼓励引导民间资金成为创业资本。切实强化服务，依法保护投资者的合法权益。

（二）大力加强基础设施建设，进一步夯实发展基础

加强交通基础设施建设。全年完成交通建设投资30亿元。建成S204南茅公路复线，完成县到乡镇公路水泥（油）路面改建170公里，完成通畅工程水泥路面改建1330公里。加快推进益宁城际干道、益阳绕城高速、二广高速益阳段及桃马联络线、杭瑞高速益阳段及大通湖联络线、S308西姚公路和黄柘公路、桃江至横市二级公路、益阳至芦林潭千吨级航道、益阳港千吨级码头等在建项目建设。开工建设石长铁路复线益阳段、S225安化段二级公路、沅江黄茅洲大桥、沙头（小河口）资江大桥等项目。争取洛湛铁路复线益娄段、益阳至马迹塘高速公路等年内开工。加快益阳至娄底高速公路、S205武潭至良荆界二级公路等项目前期工作进度，配合做好安（康）张（家界）衡（阳）等途经我市铁路的前期工作，争取尽快启动长益常城际轻轨前期工作。

加强水利基础设施建设。不断提高防汛抗灾能力。全年完成水利建设投资6亿元。抓好洞庭湖综合治理、水利工程结合血防灭螺项目，启动大通湖东垸、共双茶垸、民主垸应急安全建设，推进大型泵站更新改造。加强农田水利设施建设，完成56座病险水库除险加固工程，抓好大型灌区续建配套和节水改造、重点中小流域治理，加强粮食主产区末级渠系建设。做好山洪地质灾害防御工作。抓好农村饮水安全工程建设，解决15万人的饮水安全问题。

加强能源基础设施建设。大力推进桃花江核电站的前期准备工作，争取国家早日核准开工建设，并以此为契机，搞好相关产业的开发。加快白竹洲水电站等项目建设步伐。力争金塘冲水利枢纽工程年内开工建设。尽快落实沅江漉湖等风电项目的投资主体，争取国家支持。加快完善城乡电网，加大对农村特别是偏远地区电力设施建设的投入力度，新建灰山港220千伏变电站、复兴变电站至益阳东变电站220千伏双回线路。

加强信息基础设施建设。充分发挥信息产业在扩大内需、促进经济增长中的作用，大力推进信息化与工业化融合。推进企业和农村信息化建设，建好2个信息化示范企业、10个信息化示范乡镇、300个信息化示范村，农村宽带网络覆盖率达到70%。新建益阳移动通信指挥中心枢纽大楼，实施村村通信工程，建设移动基站300个，实现农村地区移动网络全覆盖。加快推进数字电视整体转换，建设县市光缆干线传输网络，积极开发数字多媒体移动电视系统，继续抓好广电“村村通”工程。加快电子政务建设。

（三）打造以高新区东部新区为龙头的工业园区体系，全力推进新型工业化

加快工业园区建设。进一步加快高新区东部新区建设，打造我市承接产业转移核心区、两型社会建设试验区和统筹城乡发展示范区。创新发展机制，按照“特事特办、先行先试”原则，下放管理权限，实行封闭式管理，做到“园区问题在园区解决，园区事务在园区办结”。抓好东部新区基础设施建设，力争完成起步区12平方公里的主要道路和综合服务区建设。继续抓好高新区南扩区的发展。以基础设施配套为重点，抓好区县（市）工业园和重点乡镇工业小区建设，提升各类园区的承载功能。

支持企业做大做强。认真落实扶持企业发展的各项政策。继续推进国有企业改革，引导企业完善法人治理结构。立足资源优势和产业基础，鼓励引导企业通过兼并重组、股份制改造、引入战略合作伙伴等多种途径加快发展，集中打造一批优势产业，做大做强做优一批骨干企业。全面实施“中小企业成长工程”，培育一批“小巨人”企业。完善领导联系重点企业责任制，加强企业服务体系建设，积极开展“支帮促”活动，切实帮助企业解决融资、用地、能源、运输等方面的问题，为企业发展营造良好的环境。

提高自主创新能力。充分发挥科技的支撑引领作用。围绕“引进、合作、服务”主题，以产业科技创新为主线，以引进、消化、吸收、再创新为主要路径，推进产学研结合。加快科技创业服务中心和孵化器建设，加强高科技成果应用示范，推进科技成果产业化。建立高新技术产业培育机制，大力发展高新技术企业。实施名牌和标准化战略，培育一批国、省名牌产品和驰（著）名商标，支持安化黑茶、艾华电子、辣妹子公司等参与行业和国家标准制定，努力形成支撑益阳工业发展的品牌体系。

大力推进全民创业。进一步激发全社会创业活力。大力支持非公有制经济发展。认真落实以创业促就业的政策，降低创业门槛，鼓励支持高校毕业生自主创业、外出务工经商人员返乡创业。在各级各类工业园区逐步开辟创业基地，打造创业平台。建立创业项目库，筛选一批好的创业项目定期发布。实行市县乡三级联动，大力开展创业培训。成立创业指导服务中心，加快发展各类创业中介服务机构，为创业者提供信息、技术、人才、会计、法律等系列服务。大力弘扬艰苦创业精神，在全市开展优秀创业者评选活动。努力创造条件，让一切投资创业者安心创业、放手发展，让全社会的创业活力充分迸发、财富充分涌流。

（四）大力发展现代农业，扎实推进社会主义新农村建设

推动土地承包经营权流转。在加强耕地保护、稳定土地承包关系、保障农民土地收益的前提下，根据依法、自愿、有偿的原则，加快农村土地承包经营权流转，推动农业规模经营，促进现代农业发展。制定鼓励扶持政策，建立农村土地承包经营权流转中心，完善流转保障机制，鼓励农民采取转包、入股、出租等方式流转土地承包经营权，促进土地向专业基地集聚，向科技能手集中。

推进农业产业化经营。按照高产、优质、高效、生态、安全的要求，加快转变农业发展方式，大力发展生态农业、设施农业、立体农业和休闲农业。抓好五大农业产业链的一体化经营。有效遏制耕地抛荒，稳定发展粮食生产。扶持黑茶产业做大做强，继续抓好水果、蔬菜、水产等产业建设，推动生猪产业持续健康发展。高标准建设一批区域化、规模化、特色化农产品基地。以推进精深加工、延伸产业链条为重点，培育发展一批农产品加工龙头企业。继续支持各类农民专业合作组织发展，完善公司加基地带农户的产业化经营模式。

完善现代农业服务体系。抓好优良品种、先进适用技术的推广。采取有效措施，加强新型农机的研发和应用，提高农业机械化水平。强化动植物疫病防控和农产品质量安全监管，全面实施农产品市场准入制度，提高农业安全发展水平。大力推动标准化生产，积极开展产地认定和产品认证工作。抓好农产品交易市场建设，搞活农产品流通。积极推进农业保险，防范农业风险。加强农民培训，培育高素质的现代农业经营主体。

抓好新农村建设基础工作。坚持统筹城乡发展，按照城乡基础设施同建、公共服务共享的要求，着力推进城乡基本公共服务均等化。加大对农业的投入，落实各项惠农政策，减轻农民负担。深化农村综合改革，积极推进县乡财政体制和农村社会保障、集体林权、金融、户籍制度等改革。抓好村容村貌整治、乡风文明建设和农村民主管理，加强乡村清洁工程建设。认真开展大通湖统筹城乡发展试点和新农村示范片、示范村建设，探索新农村建设的不同模式。继续加大对库区、老区和贫困地区开发的扶持力度。加强村级班子建设，落实村干部待遇。

（五）努力加快城镇发展，提升新型城镇化水平

抓好城镇规划。加大规划投入。加快城镇控规编制，实现中心城区和东部新区近期建设区控制性规划全覆盖，完成中心城区主要专业规划和村庄整治规划编制。按照建设宜居山水生态城市的规划理念，保护好山体水体。加强规划审批与管理，开放规划展示馆，提高规划透明度。严格规划执法。按照属地管理原则，建立以区为主、市区协同配合的长效执法机制，强化县（市）执法职能，实行经常性监管与专项行动相结合，坚决制止违章建筑，依法加大拆违力度，确保规划的严肃性和权威性。

加强城镇建设。坚持“量力而行，扎实推进，突出重点，综合配套”的方针，以中心城区和县城建设为重点，抓好城镇基础设施建设。突出加快中心城区发展。推进资阳区旧城改造、城市防洪、火车站周边改造提质、城北污水处理厂等重大项目建设；完成桃花仑路、金山北路等道路改造；继续推进人行道、路灯、地下管网、消防设施、公厕、公交站亭的配套和背街小巷的提质。坚持以植树为主，大幅度提高城市绿化水平。抓好“城中村”改造。支持县城及重点镇增强城镇功能，抓好沅江市、南县、桃江县、安化县等污水处理厂建设。支持一批发展基础好、辐射功能强的建制镇加快成长，将小城镇打造成乡镇招商引资和全民创业的平台。强化建设工程质量监督管理。加大政策调控力度，确保房地产业平稳健康发展。

强化城镇管理。把大力提升城市管理水平作为城市提质的一件大事来抓。坚持在从严管理上下功夫、在长效机制上作文章，努力实现城镇管理向现代化、人性化、精细化管理转变。切实抓好城镇社区建设，充分发挥社区在城镇管理中的重要作用，完善分级负责、分块包干的管理办法，落实“门前三包”责任制。深入开展市容秩序综合整治，实现城镇管理特别是中心城区管理水平跃上一个新台阶。

搞好城镇经营。加大城市资产经营力度，完善多元投资、市场运作的发展思路，促进生产要素和创新资源向城镇快速集聚。建立多元融资体制，拓宽城建融资渠道。加快市城建投资公司发行企业债券步伐。严格实行城市经营性土地使用权拍卖出让制度，提高土地经营效益。

（六）加快发展服务业，努力扩大消费需求

加快发展生产性服务业。以优化产业配套环境为目标，突出发展现代物流业，加快益阳物流园建设，引进现代物流企业，逐步建设布局合理、装备先进、运行规范的现代物流服务体系。大力培育投资品市场。规范发展会计、审计服务等商务中介组织，积极发展人才、劳动力市场等中介组织，推广代理、代办、经纪等中介服务方式，逐步形成特色鲜明、功能完善的商务服务体系。加快海关、商检机构建设，扶持出口企业发展，不断扩大对外贸易。

大力发展旅游业。把旅游产业作为支柱产业来打造，作为新的经济增长点来培育。完善旅游规划体系，启动《益阳市旅游发展总体规划》修编工作。抓好创建中国优秀旅游目的地城市迎国检工作。继续抓好雪峰湖地质公园、安化茶马古道、梅山文化生态园、桃花江竹海、山乡巨变第一村、黄家湖风景区、南洞庭湿地风景区、黑茶文化广场等景区景点建设，打造旅游核心产品和旅游精品线路。继续办好大闸蟹美食节和捕捞节。推动旅游区域合作，强化宣传促销，扩大客源市场。加强旅游行业监管，提高旅游服务水平。

努力扩大消费需求。坚持把扩大消费作为调整经济结构、改善增长质量的主要措施，努力提高城乡居民收入，增强居民购买力。继续推进“万村千乡市场工程”，抓好农村新型现代流通网络工程建设，建立沟通城乡、服务农民的商品流通体系。积极开展“家电下乡”推广活动，努力扩大农村消费。改善消费环境，培育消费热点，合理引导发展汽车、文化、体育健身等新兴消费领域，推动消费结构升级。

（七）认真抓好两型社会建设，全面建设绿色益阳

加强环境保护。认真实施《建设绿色益阳行动纲要》。实行环境保护一票否决制。把好新上项目环评审批关，确保建设项目环评审批率达到100%。建设市污染源监控中心，加强对重点区域和污染源的在线监测和专项治理。突出抓好资水和洞庭湖流域的环境保护。支持企业加大环保投入。抓住政策机遇，努力争取国家环保投资。开展绿色企业、绿色学校、绿色社区、绿色家庭等主题创建活动。加强农村环境保护，建设一批生态优美村镇。

促进资源节约。加快淘汰落后产能，加强重点用能企业节能管理。抓好省百家企业重点节能技改项目的实施，推进资源综合开发和利用，抓好建筑节能、交通节能和公共机构节能。大力推进城乡建设用地增减挂钩，破解经济社会发展的土地资源瓶颈制约。规范土地市场管理，促进集约用地。大力发展循环经济，构建资源循环式利用、企业循环式生产、产业循环式组合、社会循环式消费的良性互动发展模式。在抓好省级循环经济试点的同时，确定5个园区、50家企业开展循环经济试点。

抓好生态建设。着力抓好国家现代林业示范市建设，逐步形成完备的生态体系、发达的产业体系和繁荣的生态文化体系。大力植树造林，创新营林造林机制，大力发展非公有制造林，着力推广全民造林。坚持以工业化的理念抓好林业产业化建设，突出发展竹产业、浆纸板材产业、油茶产业、种苗花卉产业、森林生态旅游产业。强化流域水土保持综合治理。加强地质环境监测，重视灰山港等重点区域的地质灾害治理。开展矿山整治，坚决关闭破坏生态、污染环境的矿山。强化水功能区及水源地保护，重点保护好33个省定饮用水源。启动中心城区第二水源地的规划建设。

（八）全面发展各项社会事业，坚持不懈地改善民生

坚持以建设教育强市统揽教育工作全局。大力发展各级各类教育，突出抓好九年义务教育和职业教育。完善教育经费保障机制，确保预算内教育经费占财政支出的比例同口径提高2个百分点。健全覆盖城乡的学生资助体系。实施名师工程和教师继续教育工程，下大力气优化教师队伍结构。积极创造条件，落实教师的政策性待遇。加快国基职业教育园的建设和发展。推进中小学合格学校建设，继续深化教育教学改革，促进教育均衡协调发展。

完善公共卫生和医疗服务体系。加强农村卫生基础设施建设，健全新型农村合作医疗制度和疾病预防控制体系。强化计划免疫、社区医疗服务，切实加强血吸虫病、职业病和重大传染病的防治工作。严格卫生监督执法，加强行业管理和医德医风建设，努力让群众得到安全、有效、价廉的医疗服务。推进医疗卫生改革。积极开展爱国卫生运动。

加快发展文化体育事业。坚持先进文化前进方向，加强社会主义核心价值体系建设，大力发展和谐文化，激发文化创造活力。加快建设城乡公共文化服务体系，丰富群众的文化生活。加强网络文化建设和管理，为青少年健康成长创造良好社会环境。构建以现代传媒、新闻出版、文化娱乐为主导的文化产业体系。进一步办好《益阳日报》、益阳广播电视台和政府门户网站。广泛开展全民健身活动，积极备战2010年全省第十一届运动会。

继续稳定低生育水平。强化基层基础工作，改善农村计划生育服务条件，加强流动人口计生管理服务，兑现奖励扶持政策，综合治理出生人口性别比偏高等问题，加大社会抚养费征收力度，降低出生缺陷发生率，提高出生人口素质。

切实抓好扩大就业和社会保障工作。实施积极的就业政策，健全公共就业服务体系和就业援助制度。重点推进返乡农民工、高校毕业生、零就业家庭、城镇新增就业人员等群体的就业。切实加大就业培训力度，提高各类劳动者素质和技能水平。加强劳动执法监察，依法维护劳动者权益。加强社会保障体系建设。全面启动有城镇户籍的居民参加养老保险。扩大城镇职工养老保险和基本医疗保险覆盖面，努力提高非公有制经济从业人员、灵活就业人员的参保率，推进失业、工伤、生育保险，解决困难企业职工和关闭破产企业退休（养）人员参加基本医疗保险问题，全面巩固和发展城镇居民基本医疗保险。加强社会保险基金管理，确保基金安全和保值增值。健全城乡最低生活保障制度，做到应保尽保。建立医疗救助制度。加大廉租房建设力度，继续抓好农村安居工程和城镇经济适用房建设。发挥住房公积金制度的住房保障作用。做好洞庭湖区专业捕捞渔民解困工作。认真落实对被征地农民的安置补偿政策。积极发展社会慈善事业，重视发挥红十字会、慈善总会等社会救助团体的作用，抓好益阳市社会福利服务中心、流浪儿童救助保护教育中心和益阳市救灾备灾仓库建设，建好12所乡镇敬老院、40所村级“五保之家”。继续为民办实事，让人民群众持续获得更多看得见、摸得着的实惠。

确保社会大局稳定。认真解决人民群众利益诉求问题，建立和完善公开办信、领导接访、信访督办等制度，加大重信重访专项治理和复查复核工作力度，重点解决涉及国企改制、征地拆迁等方面的信访问题。推进“平安益阳”建设，抓好法制宣传教育和依法治理工作，强化社会治安综合治理，加强基层警务工作，健全社会治安和公共安全防控体系。整顿治安重点地区和突出问题，严厉打击各类违法犯罪和暴力恐怖活动。提高全民国家安全意识，坚决防范和打击敌对势力的渗透颠覆活动。坚持安全发展。重视和抓好产品质量安全工作，强化食品、药品等重点产品的监管。加强价格监测监管和价格预警。严格落实安全生产政府监管责任和企业主体责任。深入开展煤矿、非煤矿山、烟花爆竹、危险化学品、道路交通、建筑施工、人员密集场所等重点行业和领域安全生产隐患排查治理，落实消防安全责任，加大行政执法力度，严厉查处各类安全生产违法违规行为，有效遏制重特大事故发生。加强应急管理工作，提高应对突发事件能力。

加强国防动员和后备力量建设，加强武警部队建设，深入开展拥军优属、军民共建活动。广泛开展群众性精神文明创建活动，大力弘扬民族精神、时代精神和益阳精神。支持工会、共青团、妇联等群团组织依照法律和各自的章程开展工作、参与社会管理和公共服务。继续做好档案、统计、民族、宗教、外事、侨务、对台、科协、老龄、残疾人、地方志、人防、地震、气象、水文、无线电管理等工作。

（九）以提高执行力为重点，切实加强政府自身建设

大力提高政府执行力。全面开展提高执行力主题活动。强化执行意识，使执行成为一种纪律、一种作风、一种素养，做到接受任务不讲借口，完成任务不打折扣。雷厉风行，勇于担当，争创一流，优质高效地执行好每一项决策部署和工作任务。加强公务员队伍建设，鼓励政府公务员加强学习，提高政治理论素养、经济理论水平和业务工作能力。着力强化检查督办机制、激励约束机制，加强绩效考核，严格责任追究，为提高政府执行力提供坚强保证。

全面推进依法行政。认真贯彻落实《国务院关于加强市县政府依法行政的决定》和《湖南省行政程序规定》，严格按照法定权限和程序行使权力、履行职责。认真落实依法行政工作情况报告制度。完善行政决策机制，健全重大问题集体决策制度、专家咨询制度、社会公示和听证制度，强化行政决策责任追究，确保决策民主化、科学化。全面落实行政执法评议考核制度，规范行政执法行为。

切实转变政府职能。围绕建设服务型政府，创新政府管理，全面放松对经济的管制，切实加强对经济的服务。进一步加强行政效能建设，提高办事效率。进一步精减审批项目，规范审批行为；进一步简化办事程序，缩短办结时间；进一步加强各级政务服务中心建设，大力推行政务公开，提高政府工作透明度，为投资者和人民群众提供优质服务。

自觉接受各方面监督。认真贯彻《政府信息公开条例》，完善政府信息公开制度和新闻发布制度。认真执行人大及其常委会的决议、决定。自觉接受人大的法律监督、工作监督和政协的民主监督，自觉接受各民主党派、工商联、无党派人士的监督，自觉接受社会监督和舆论监督。加强行政监察和审计监督。切实推进机关作风建设，弘扬艰苦奋斗、勤俭节约的优良传统。坚持不懈地抓好廉政建设和反腐败斗争，确保人民赋予的权力始终为人民谋利益。

各位代表！科学发展、后发赶超是全市人民的共同意志，是时代赋予的历史使命。让我们紧密团结在以胡锦涛同志为总书记的党中央周围，高举中国特色社会主义伟大旗帜，坚持以邓小平理论和“三个代表”重要思想为指导，深入贯彻落实科学发展观，在中共益阳市委的坚强领导下，紧紧依靠全市人民，解放思想，万众一心，开拓进取，奋发图强，为实现益阳经济社会又好又快发展而努力奋斗！
